# *In-silico* experiments of zebrafish behaviour: modeling swimming in three dimensions

**DOI:** 10.1038/srep39877

**Published:** 2017-01-10

**Authors:** Violet Mwaffo, Sachit Butail, Maurizio Porfiri

**Affiliations:** 1 Department of Mechanical and Aerospace Engineering, New York University Tandon School of Engineering, Brooklyn, NY, USA; 2 Department of Mechanical Engineering, Northern Illinois University, DeKalb, IL, USA

## Abstract

Zebrafish is fast becoming a species of choice in biomedical research for the investigation of functional and dysfunctional processes coupled with their genetic and pharmacological modulation. As with mammals, experimentation with zebrafish constitutes a complicated ethical issue that calls for the exploration of alternative testing methods to reduce the number of subjects, refine experimental designs, and replace live animals. Inspired by the demonstrated advantages of computational studies in other life science domains, we establish an authentic data-driven modelling framework to simulate zebrafish swimming in three dimensions. The model encapsulates burst-and-coast swimming style, speed modulation, and wall interaction, laying the foundations for *in-silico* experiments of zebrafish behaviour. Through computational studies, we demonstrate the ability of the model to replicate common ethological observables such as speed and spatial preference, and anticipate experimental observations on the correlation between tank dimensions on zebrafish behaviour. Reaching to other experimental paradigms, our framework is expected to contribute to a reduction in animal use and suffering.

Experimental studies with animal subjects face ethical issues that demand the exploration of alternative approaches to reduce the number of subjects, refine experimental designs, and replace the use of live animals (3Rs)^1^. While animal studies are the ultimate cornerstone against which new hypotheses should be tested, computer simulations, or *in-silico* experiments, constitute a powerful tool in preclinical research. *In-silico* experiments could be used to: (i) gather preliminary evidence in favour or against a specific hypothesis through systematic pilot trials on the computer^2^; (ii) achieve statistical power in an animal study via careful selection of the number of trials, independent and dependent variables, and frequency of measurement^3^; and (iii) reinforce the scientific merit of existing observations through comparison against synthetic data and statistical analysis informed by modelling insight^4^,^5^,^6^,^7^.

In recent years, we have witnessed a surge in the number of *in-silico* experiments, including molecular biology^8^, genetic assays^9^, tumour growth^10^, dermatology^11^, bone remodelling^12^, organ failure^13^, and toxicology^14^. These studies have helped build thrust in the potential of *in-silico* experiments and develop a strong link between simulations and experiments, which are more often coupled in new preclinical studies. However, the use of computer simulations in behavioural neuroscience has been limited, partly due to the complexity of animal behaviour and partly because of the unavailability of refined three-dimensional (3D) data needed to calibrate and validate mathematical models underlying such simulations^15^,^16^,^17^,^18^,^19^,^20^.

Although animal research has mainly focused on rodents^21^ and primates^22^, zebrafish (*Danio rerio*) are attaining popularity for both technical and economic reasons. Zebrafish are genetically similar to humans, especially with respect to diseases^23^,^24^, where a strong genetic similarity is found^25^. Zebrafish embryos and larvae are transparent, which afford the possibility of monitoring inflammatory processes and indicators of disease without surgery^24^. Further, unlike mammalian animal models, zebrafish can be bred in large numbers^26^, have a short inter-generation time^27^, and can be stocked at high densities^24^. These factors are fuelling intensive research in zebrafish behavioural phenotyping, whereby locomotory patterns are continuously being identified and categorized to create a comprehensive catalogue to aid in measuring zebrafish behaviour^28^, for use in neurobehavioural research.

A large number of experimental protocols have been proposed to investigate zebrafish behaviour, often borrowing from murine models. For example, classical choice^29^, place preference^30^, open field^31^, and light/dark preference tests^32^ have been successfully used to investigate the neurobiological basis of social behaviour, study fear and anxiety, and explore the effect of psychoactive compounds and genetic manipulations^33^,^34^,^35^,^36^. In these experiments, zebrafish behaviour is measured in terms of locomotory patterns that are typically scored from labour intensive manual observations and seldom using automated computer tracking. Only recently, technical breakthroughs in automated tracking have enabled the complete reconstruction of zebrafish swimming in 3D^17^,^37^,^38^,^39^,^40^. *In-silico* experiments may significantly contribute to the design of new test batteries, by enabling the systematic analysis of dependent and independent variables while isolating key experimental factors that may act as confounds during testing. Further, *in-silico* experiments may be used to identify false positives and negatives, by providing an empirically-based ground truth on which to contextualize new experimental observations^41^,^42^.

The first computational model of zebrafish swimming has been proposed by our group in refs 43, 44, 45 and 46. In this series of studies, we have proposed a data-driven modelling framework to predict the turn rate dynamics and speed modulation of zebrafish swimming in a shallow water tank. To capture the burst-and-coast swimming style of zebrafish, where sporadic bursts of the tail are followed by coasting phases without tail beating^47^,^48^, we have proposed the use of jump processes, often employed in financial engineering to model extreme events in the time evolution of assets prices^49^,^50^. Specifically, in refs 43, 44, the turn rate dynamics of zebrafish is described through a mean reverting stochastic process that incorporates jumps. The jump persistent turning walker (JPTW) accounts for the sudden and fast turns observed in the yaw rate of zebrafish swimming. In ref. 46, we have contemplated the possibility of speed modulation during in-plane manoeuvring, where fish slow down their motion when turning. This modelling framework has been recently extended to study social behaviour in ref. 45 by demonstrating the role of speed modulation in regulating collective dynamics of a shoal of zebrafish. Using a similar approach based on stochastic processes, a recent study^51^ has simulated zebrafish orientation within a kinematic model, by sampling from a probability density function that accounts for a spherical visual perception field. These models, built and calibrated on 2D datasets, are expected to suffer from the same limitations that are attributed to 2D behavioural analysis, including unrealistic representation of swimming activity and novelty seeking behaviour^17^; incomplete characterization of stress-related behaviours, such as thigmotaxis and geotaxis^28^; and limited comparability of different experimental assays due to unmeasured variations in depth^17^.

Here, we present a new 3D model for zebrafish swimming, extending our previous work to three dimensions and encapsulating wall interaction and speed modulation. We model both yaw and pitch rates as JPTW, and we treat speed as a mean reverting square process^52^. Wall avoidance is modelled as a decaying function of time to collision^53^,^54^, and is further adapted to include collision avoidance at high speeds. Model parameters are calibrated with 3D trajectory data of adult zebrafish swimming in a parallelepipedal tank with a rectangular base for 10 minutes^38^. Model validation is performed by conducting a simulation study and comparing speed and indirect behavioural measures, such as positional preference along the horizontal and vertical directions, with experimental observations. To further evaluate if our model can be used to contribute to replacement and reduction, we conduct a second simulation study, investigating the effect of tank dimensions on zebrafish behaviour. We systematically vary the tank dimensions for 60 combinations of length, width, and height, and compare our results with data collated from the literature. We demonstrate the use of *in-silico* experiments in a prospective study, which would require thousands of subjects and months of experiments to explore the parameter space utilized in our numerical simulations.

## Results

### First *in-silico* experiment for model calibration

**Figure 1:**
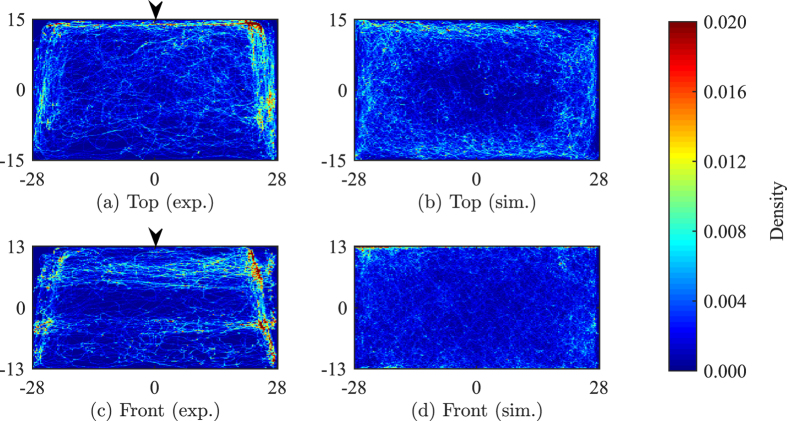
Position distribution of nine experimental (**a** and **c**) and 10 simulated (**b** and **d**) fish, along the length (**a** and **b**) and the water column (**c** and **d**). The bin size is 0.28 × 0.15 cm for the top view and 0.28 × 0.13 cm for the front view. The density is saturated at 0.02 to ease the visualization of the data, although the maximum value is 0.07. Arrows indicate the tank sides not covered with white contact paper.

Position distribution from the top view over all of 10 trials show that fish explore all sections of the tank with a marked preference for the corners and side walls of the tank (Fig. 1(a)). The lack of symmetry in the density along the tank width is related to the fact that the front portion of the tank is not covered with contact paper to enable tracking using the front camera. The front view indicates a similar exploratory behaviour along the depth of the tank (Fig. 1(c)) with more time spent near the side walls. Model predictions yield a response that is qualitatively similar to experimental observations, with larger density toward the walls (Fig. 1(b)).

However, the model does not anticipate the wall preference observed in the experimental data. Different from experiments, where fish tend to prefer the clear side of the tank to the side covered with contact paper, simulation results show no such preference for a particular side (Fig. 1(d)). This is because the model implements the same interaction rule on each side. Along the front view simulation results do not reproduce preference to the smaller sides or to the water surface which is different from tank bottom (Fig. 1(d)).

**Figure 2:**
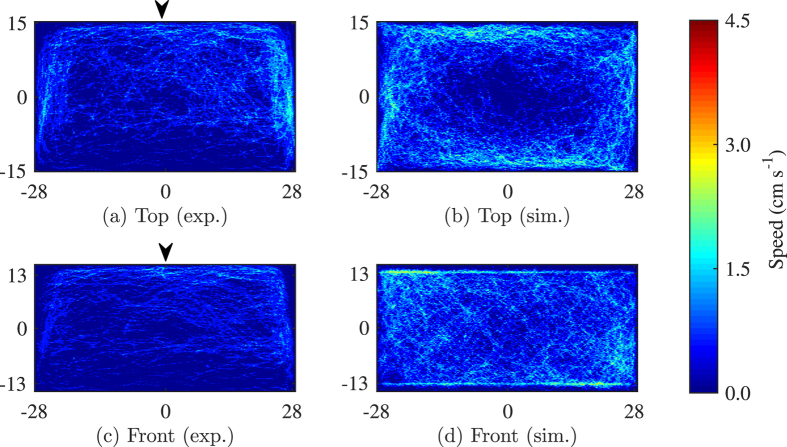
Speed distribution of nine experimental (**a** and **c**) and a 10 simulated (**b** and **d**) fish, along the length (**a** and **b**) and the water column (**c** and **d**). The bin size is 0.28 × 0.15 cm for the top view and 0.28 × 0.13 cm for the front view. The speed is saturated at 4.5 cm s^−1^ corresponding also to the maximum averaged speed value. Arrows indicate the tank sides not covered with white contact paper.

**Figure 3:**
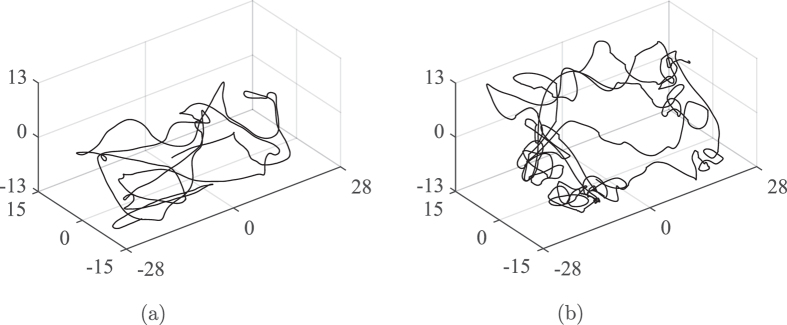
Sample trajectories of experimental (**a**) and simulated fish (**b**) in 3D for two minutes.

Experimental results show that speed distribution within the tank is asymmetric along the tank plane (Fig. 2(a)) with fish swimming at a higher speed near the side walls and the front wall, which is open for the camera to record. An asymmetry in experimental data is also visible along tank depth with higher speeds near the water surface (Fig. 2(c)). Simulation results (Fig. 2(b) and (d)) are in agreement with experimental observations, whereby they predict larger speeds in the vicinity of the walls. Different from experiments, simulation results do not differ along the front wall, as the model implements the same interaction rule on each wall. In terms of individual motion characteristics, Fig. 3 compares a sample trajectory of a live fish with a simulated fish, offering evidence for the model ability to replicate the sharp turn associated with avoidance manoeuvres and the fragmented tracks within the bulk of the tank.

**Figure 4:**
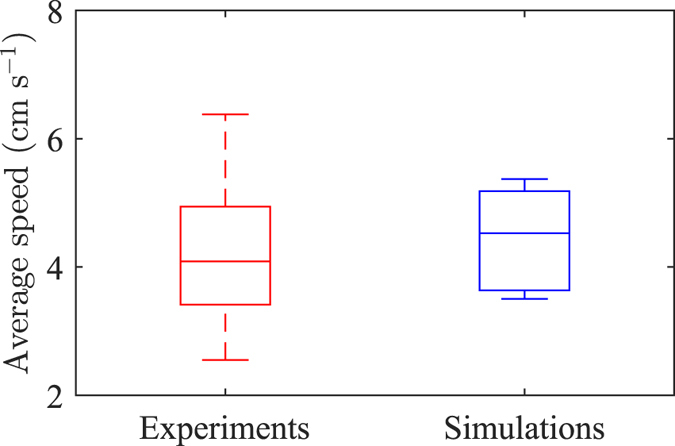
Comparison of average speed between live and simulated fish. Experimental data refer to Table 1 in ref. 38. The bottom and top of each box are the first and third quartiles, the band inside the box is the median, and whiskers identify one standard deviation above and below the mean.

**Figure 5:**
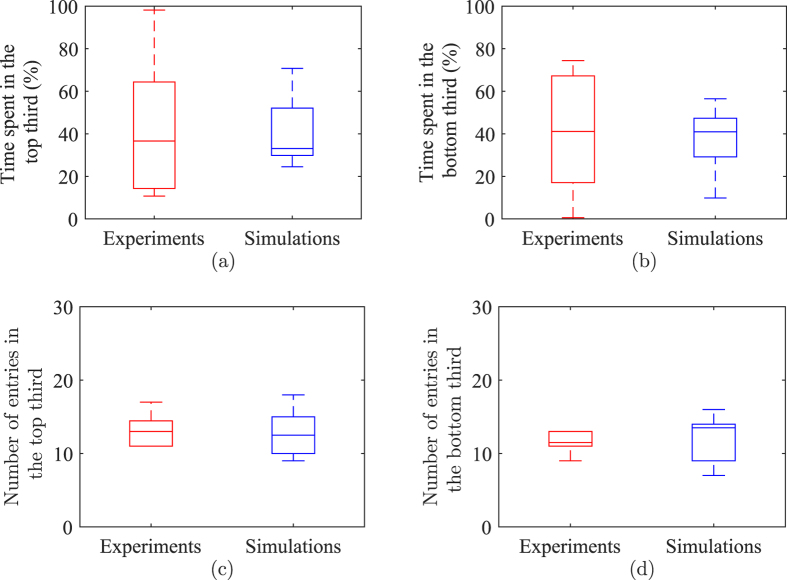
Comparison of fish positional preference along the water column between live and simulated fish: time spent in the top third (**a**); time spent in the bottom third (**b**); number of entries in the top third (**c**); and number of entries in the bottom third. Experimental data refer to Fig. 7 of ref. 38. The bottom and top of each box are the first and third quartiles, the band inside the box is the median, and whiskers identify one standard deviation above and below the mean.

**Figure 6:**
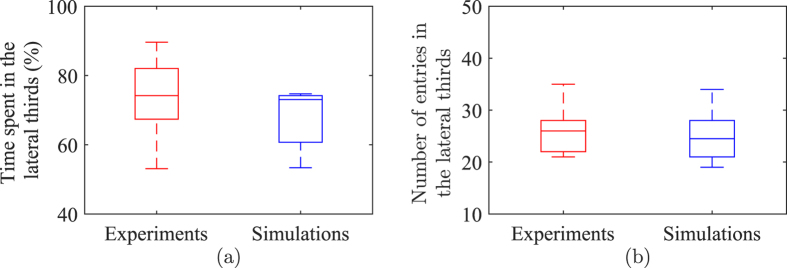
Comparison of fish positional preference along the length of the tank between live and simulated fish: time spent in the lateral sides of the tank (**a**) and number of entries in the lateral sides (**b**). Experimental data refer to Figs 6 and 7 of ref. 38. The bottom and top of the each are the first and third quartiles, the band inside the box is the median, and whiskers identify one standard deviation above and below the mean.

Figures 4, 5 and 6 compare the behavioural response between live and simulated fish using a non-parametric Kruskal-Wallis test to examine the null hypothesis that the values are sampled from different distributions with different median. Figure 4 displays experimental observations and model predictions on fish activity, measured through the average speed. Specifically, we find that model predictions are not significantly different from experimental data (*χ*^2^(1) = 0.8229, *p* = 0.3643).

Figure 5 presents a detailed comparison between experimental data and model predictions on fish spatial preference along the water tank. The model is successful in anticipating the time budgeting along the water column, whereby simulation data and experiments are similar (time spent in the top third: *χ*^2^(1) = 0.0229, *p* = 0.8798 and time spent in the bottom third: *χ*^2^(1) = 0.0229, *p* = 0.8798). The number of entries predicted by the model is also statistically equivalent to experimental observations (number of entries in the top third: *χ*^2^(1) = 0.1169, *p* = 0.7324 and number of entries in the bottom third: *χ*^2^(1) = 0.1450, *p* = 0.7033).

Finally, Fig. 6 offers validation for the model capability to anticipate fish positional preference along the length of the tank. In line with all the previous comparisons, model predictions are found to be statistically indistinguishable from experimental data (time spent in the lateral sides: *χ*^2^(1) = 1.12, *p* = 0.2899 and number of entries in the lateral sides: *χ*^2^(1) = 0.2808, *p* = 0.5961).

### Second *in-silico* experiment for model evaluation

**Figure 7:**
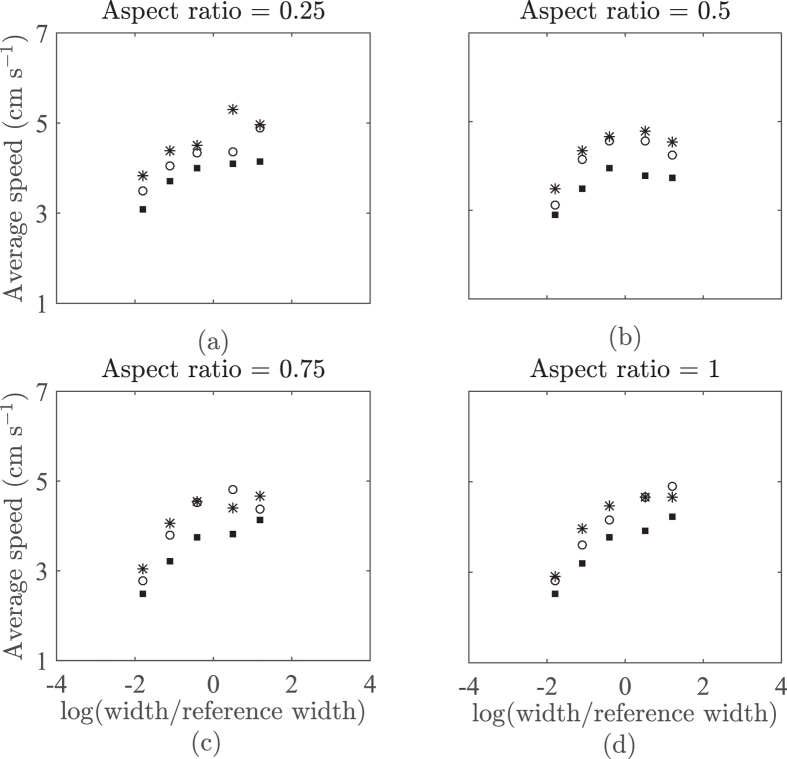
Simulated fish speed by varying tank depth and width with aspect ratio 0.25 (**a**), 0.5 (**b**), 0.75 (**c**), and 1. The symbols star, dot, and square represent tank depths of 10, 20 and 30 cm, respectively. The function log(•) refer to the natural logarithm and the width is divided by the width of the experimental tank in ref. 38, which is taken to be the reference width.

**Figure 8:**
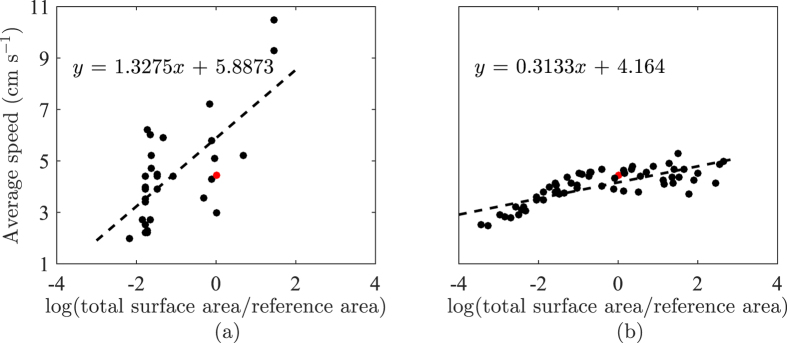
Effect of the total tank surface on fish speed obtained from (**a**) data compilation from literature, and (**b**) *in-silico* experiments of zebrafish swimming in an open field tank. The function log(•) refer to the natural logarithm, the total surface area is divided by the surface area of the experimental tank in ref. 38, which is taken to be the reference area. For reference, we display in red the point corresponding to the experimental data in ref. 38 used to calibrate the model.

Our simulation results presented in Fig. 7 suggest that fish speed is an increasing function of the tank planar dimensions across different depths. Specifically, using a GLM analysis, fish speed is correlated to tank width for all the tested aspect ratios, namely, 0.25 (Dispersion = 0.160, *F*_1,13_ = 15.8, *p* < 0.01), 0.5 (Dispersion = 0.247, *F*_1,13_ = 6.49, *p* = 0.0243), 0.75 (Dispersion = 0.222, *F*_1,13_ = 19.8, *p* < 0.01), and 1 (Dispersion = 0.145, *F*_1,13_ = 41.6, *p* < 0.01). By aggregating the simulation data with respect to the total surface area (Fig. 8), we find a strong correlation between the logarithm of the total surface area and the average speed (Dispersion = 0.168, *F*_1,58_ = 94.4, *p* < 0.01). By performing the same analysis on experimental data from Table 1, we uncover a similar, albeit weaker, trend between the average speed and the total surface area (Dispersion  = 2.01, *F*_1,30_ = 27.4, *p* < 0.01). Although the GLM analysis shows a higher dispersion in experimental data compared to simulation results, the slope is also steeper. Thus, experimental data indicate the presence of a stronger effect than computational predictions.

## Discussion

In cellular and molecular biology, mathematical models of regulatory networks, supported by high-throughput data collection, have enabled the seamless integration of computer simulations and experimental research^8^,^11^,^12^,^13^,^14^. Benefits of an *in-silico* approach include: the possibility of setting up advanced starting points for testing novel hypotheses; speeding up the validation of challenging propositions; and putting forward a direct, tangible opportunity to reduce the extent of animal use. A necessary step toward bringing such benefits to the field of animal behaviour is the formulation of mathematical models that can faithfully reconstruct animal locomotion of individuals^53^, which in turn better informs collective motion models^55^,^56^,^57^. In the case of zebrafish, a model organism for preclinical studies, a comprehensive catalogue of observed behaviours^28^ and high-volume datasets from the literature^17^,^37^,^38^,^39^,^40^ make a compelling case for the development of a framework to support *in-silico* experiments.

An accurate model that is able to robustly reproduce the full catalogue of zebrafish behaviours is far from accomplished. Instead, we describe here a model that conforms to basic zebrafish locomotory patterns in 3D, laying the foundations for *in-silico* zebrafish experiments. We extend our previous approach^43^,^44^ of using jump diffusion stochastic processes to reproduce segments of zebrafish turning motion in 2D to a more realistic scenario, where fish swim in 3D adjusting their speed and interacting with the tank walls throughout an entire experiment. Comparison with experimental data demonstrates that our model is successful in qualitatively reproducing zebrafish swimming behaviour, and quantitatively predicting activity and positional preference, in terms of average speed, time budgeting, and number of entries in longitudinal and transversal sections of the tank. Visual differences in positional preference and activity are likely due to complex interactions with clear tank walls, water surface, and tank bottom, as well as difference in interactions due to wall size. The model consists of 16 parameters, which are calibrated from complete experimental trajectories of zebrafish swimming in 3D. Although the parameter space is large, the identification procedure is relatively robust, as evidenced by the limited variability in model parameters across the nine subjects considered in the experiment. Also, by automatically isolating instances when fish interact with the wall, the procedure is effectively partitioned in two consequent steps, reducing the number of parameters to be determined at once. Model parameters indicate that yaw and pitch rate parameters, such as the relaxation rate and variability have similar values. This is because the fish orientation is defined in the tank inertial frame, and turning up towards the water surface sideways may be interpreted as pitching in the inertial frame. On the other hand, jump intensity in the pitching direction is higher than that in the yaw direction, which is likely caused by stress-related behaviour in experimental subjects^17^,^35^,^58^,^59^. In a novel environment, zebrafish have been shown to repeatedly change their swimming depth^40^,^60^,^61^, which in our model is associated with the jump process in the pitch direction.

The potential of the model to complement empirical research is demonstrated through *in-silico* experiments that seek to understand the effect of tank dimensions on zebrafish activity–a study which would otherwise require thousands of animals and multiple experimental setups. These computational experiments reveal that the average speed of zebrafish should increase with all of the tank dimensions. By aggregating data with respect to the overall tank surface, we find a strong correlation with the average speed. A correlation between the tank surface and average speed is also evidenced from experimental data collated from the literature, even though the slope of the correlation is different from the simulation data. While it is likely that fish activity is influenced by a number of factors that are not constant across the collated dataset, such as illumination^62^ and habituation time^63^,^64^, we could explain the observed correlation between speed and surface area through two different reasons. First, fish speed may increase due to fewer instances of boundary avoidance as the tank size increases. Second, speed may increase due to thigmotaxis^61^, produced by thrashing or escape behaviours along the tank walls and corners^28^. This stress-related behaviour may be accompanied by high accelerations^28^, which would, in turn, result into a larger average speed.

In its present incarnation, our model appears to weight the second possibility more than the first. This is also evident from the speed distribution plots of simulated data, which suggest increasing speeds in the vicinity of the side walls, bottom wall, and free surface. Indeed, our model explicitly captures fish positional preference for walls and corners in the wall interaction function. However, our model does not directly encode for the first possibility, namely increased speed due to fewer encounters. This behaviour could be directly related to increased exploratory tendency in real fish, leading to higher average speeds^59^ for larger tanks size, and thus the steeper slope observed in the experimental data. Encoding such behaviours within our model may likely be accomplished by setting model parameters such as relaxation rate or wall avoidance as a function of tank dimensions. The nature of this dependence, however, will require detailed experiments that allow an accurate and reliable calibration of the model. Future work will focus on further validating this claim through new experiments with a wider range of tank sizes, and investigating the dependency of the model on other experimental factors, such as varying illumination^62^ and habituation time^63^,^64^, which could influence the observed correlation.

An approach similar to modelling wall interaction could be utilized to describe response to external stimuli, such as drawing towards novel objects or light, avoidance of predators, alarm reactions to sudden threats, and shoaling toward conspecifics. For example, with respect to shoaling behavior, the present model would be used to represent the individual dynamics of each zebrafish, interacting as a networked dynamical system. The network of interactions would then be based on relative orientation and position, with weights associated with each cue that can be calibrated using experimental data building on existing models for fish interactions in 2D^53^,^65^. The model could also be expanded to encapsulate innate behaviours, such as home-base behaviour and habituation^64^. Home-base behaviour may be integrated into the model by including preference for the initial region of the environment that decays as a function of time, with the rate of decay calibrated on the basis of actual experiments. Habituation, which manifests as a time effect in fish behaviour as it enters a new environment^63^,^64^, could entail creating a virtual, tighter boundary within the environment that expands to the full tank size as time progresses.

The model may also be expanded to describe the effect of psychoactive compounds, through a controlled dependence of model parameters on pharmacological treatment. For example, in the context of the 2D JPTW, we have demonstrated in ref. 44 that alcohol treatment controls both relaxation rate and jump intensity. In the proposed 3D model, the effect of ethanol should also manifest in the speed modulation and wall interaction. As ethanol concentration is varied, zebrafish should modify their swimming style, since both the tail-beat amplitude and the tail-beat frequency have been shown to robustly decrease for high ethanol concentrations^66^. As a result, the parameters describing the instantaneous change in fish speed and the coupling with the turn rate dynamics should be adjusted as a function of ethanol concentration. The parameters related to the interaction with the wall should depend on ethanol concentration, whereby exposure to intermediate concentrations have been shown to reduce wall interaction, while higher concentration could influence the extent of the interaction^67^.

Creating the scientific and technical knowledge for affording comprehensive *in-silico* experiments on zebrafish will require a systems-level approach^68^. This entails not only identifying and modelling individual behaviours, but also understanding how one behaviour may lead to another. For example, habituation may lead to reduced activity and exploration, and interaction may lead to increased sociality. The proposed model should be considered as a first necessary step toward *in-silico* research. Among the several directions that will be pursued to improve model capability, zebrafish response to conspecifics is perhaps most promising, as it will require augmentation of data-driven response functions^45^,^53^ in 3D, to immediately test social behaviour.

Recent years have witnessed sustained movements that strenuously advocate the respectful treatment and welfare of animals in laboratory research. With approximately twenty million animals used in research and testing annually, policy-makers, citizens, and scientific authorities alike yearn for alternative approaches, while retaining the fundamental instrument of scientific investigation and the improvement of human health. Here, we have proposed the systematic integration of computer modelling as a new, unprecedented means to complement preclinical research on animal behaviour, focusing on the zebrafish-animal-model. While our simulations cannot replace *in-vivo* experiments, they can constitute an economical (time and budget-wise) complement to inform new experimental design and engineering approaches^69^,^70^,^71^,^72^, along with the definition of mathematically grounded measurable parameters^73^,^74^,^75^,^76^, leading to a consistent reduction of experimental subjects.

## Materials and Methods

We begin by formulating the model for the time evolution of the yaw rate, pitch rate, and speed, all of which are assumed to be coupled. Environmental interactions with the boundaries–tank walls, tank bottom, and water surface–are incorporated into the yaw and pitch rates in the form of avoidance reactions. We then detail the experimental data used to calibrate the 3D model, followed by model calibration where individual parameters are estimated using maximum likelihood estimation (MLE).

### Ethics statement

The data used in this paper are obtained from the control condition of trajectory dataset published in ref. 38. The experimental methods in ref. 38 were performed in accordance with the relevant guidelines and regulations, and were approved by the University Animal Welfare Committee of New York University under protocol number 131424, and the Animal Welfare Oversight Committee of the Polytechnic Institute of New York University (now New York University Tandon School of Engineering which was independent from New York University until 2013 with respect to animal care) under protocol number AWOC-2013-103.

### Modelling zebrafish motion in 3D

**Figure 9:**
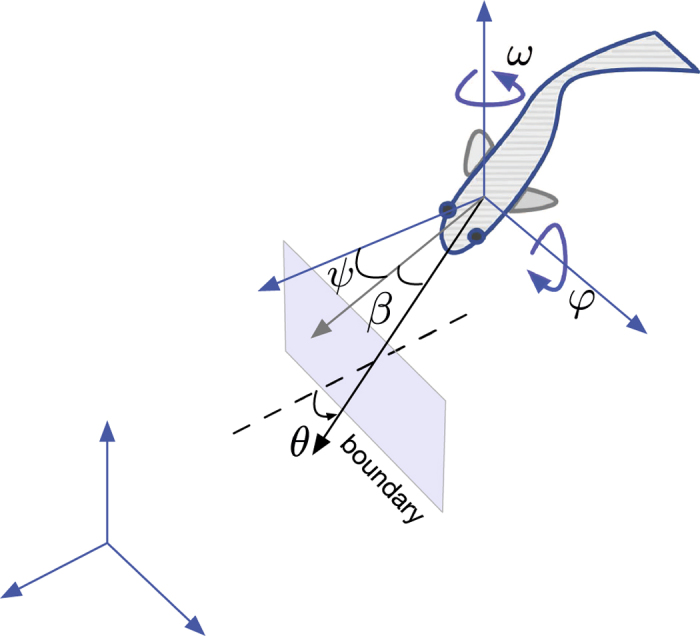
Fish orientation in terms of the azimuth (*ψ*) and elevation (*β*) in the tank inertial frame (blue). Fish yaw rate, pitch rate, and speed are modelled as stochastic processes. Fish interaction with the boundary is modelled as a function of the predicted time to collision and the angle made by the projected fish heading to the normal at the impact point *θ*.

Fish yaw rate in the tank frame (Fig. 9) is modelled as a jump persistent turning walker (JPTW), following our previous work on zebrafish 2D swimming in shallow water tanks^43^. Briefly, the instantaneous variation at time *t* of the yaw rate, d*ω*_*t*_ (rad s^−1^), is described as the sum of a deterministic term, which quantifies the deviation from a desired value, and two stochastic terms, which represent variability in the yaw rate in the form of increments of a Wiener process and intermittent large turns in the yaw direction in the form of a jump process. Specifically, the JPTW for the yaw rate reads


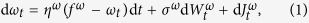


where *η*^*ω*^(s^−1^) is the relaxation rate at which the yaw tends to reach the desired value *f*^*ω*^, which encapsulates wall interaction; 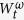 is the standard Wiener process, whose time increment is a Gaussian with variance equal to dt; *σ*^*ω*^(rad s^−3/2^) is a weighting parameter used to scale the Wiener process; and 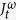 is the yaw rate jump process.

We model the wall interaction *f*^*ω*^ as a decaying function following the approach in ref. 54, and parametrize this function with the time to collision^53^. Denoting by *θ*^*ω*^ (rad) the angle between the normal to the projected wall point of impact and the fish heading vector projected on the horizontal plane, the wall interaction function is


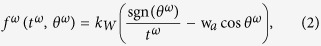


where sgn(•) is the sign function; *k*_*W*_ > 0 characterizes the intensity of the wall interaction; *w*_*a*_ (s^−1^) is a non-negative parameter that captures the tendency to follow the wall; and *t*^*ω*^ (s) is the time to collision, estimated as the time it takes for the fish to reach the wall at its current velocity. Note that the wall interaction function depends on the time *t* through both *θ*^*ω*^ and *t*^*ω*^. As such, the simulated fish turns away from the wall with higher intensity as the time-to-collision approaches zero, and for large values, the time-to-collision does not affect fish swimming. The wall interaction function (2) differs from the one proposed in ref. 53 in terms of the second summand −*k*_*W*_*w*_*a*_ cos *θ*^*ω*^, which is included to describe wall following or thigmotaxis, a commonly observed behaviour in zebrafish^28^.

Similar to ref. 43, the yaw rate jump process, 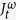, is modelled as


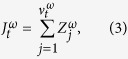


Here, 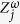, for *j* = 1, 2, …, are independent and identically distributed (i.i.d.) Gaussian random variables with zero mean and standard deviation equal to *γ*^*ω*^ (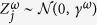) and 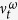 is a counting process defined such that, for time *r* ≤ *t*, 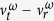 is a Poisson random variable with parameter *λ*^*ω*^(*t* − *r*). The parameters *γ*^*ω*^ (rad s^−1^) and *λ*^*ω*^ (s^−1^) measure the severity and frequency of the jumps in the yaw rate.

**Figure 10:**
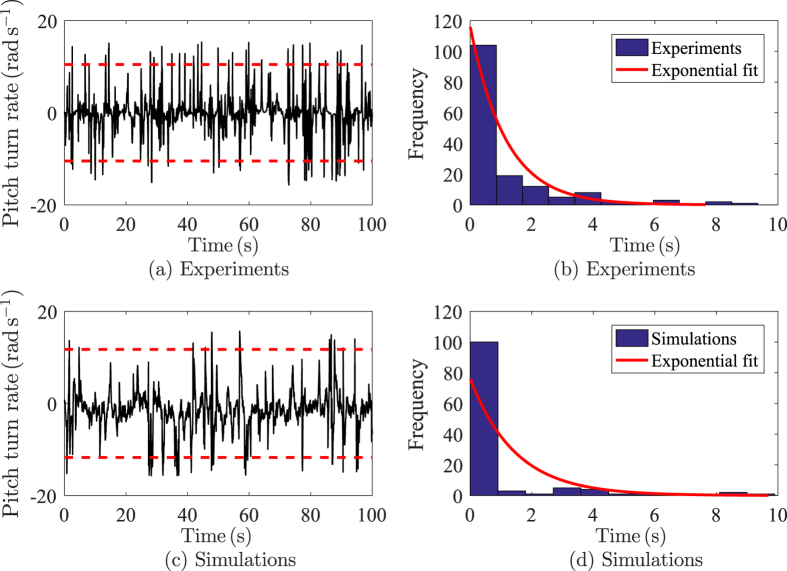
Sample experimental and simulated fish pitch rate time series, (**a**) and (**c**), and distribution of the jumps arrival time in seconds, (**b**) and (**d**). Jumps are identified with values of the pitch rate over three times the standard deviation in dashed lines (red). The arrival time is the time between two consecutive jumps, and can be fitted using an exponential distribution in solid line (red).

Fish pitch rate in the tank frame (Fig. 9) is also modelled as a JPTW, since zebrafish pitch rate can reach values beyond three times the standard deviation with arrival time following an exponential distribution (Fig. 10). The instantaneous change in pitch rate d*φ*_*t*_ is thus modelled as


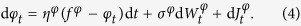


Here, *η*^*φ*^(s^−1^) is the relaxation rate in reaching the desired value *f*^*φ*^, which captures the tendency to avoid boundaries along the tank depth, that is, tank bottom surface and water surface; 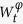 is the standard Wiener process, which is weighted by the pitch rate variability *σ*^*φ*^(rad s^−3/2^); and 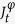 is the pitch rate jump process.

Similar to boundary avoidance in the yaw rate, boundary avoidance along tank depth, *f*^*φ*^ is parameterized by the time to collision *t*^*φ*^ and the angle between the normal to the projected boundary point of impact and the fish heading vector projected on the vertical plane oriented along the tank depth *θ*^*φ*^


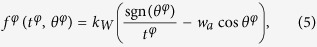


where *k*_*W*_ > 0 and *w*_*a*_ are defined in Eq (2). To reduce the number of parameters in the proposed model, we describe the interaction with the bottom wall and the free surface through the same parameters used for the side walls interaction.

Similar to the yaw, the pitch jump process is also modified to avoid collisions beyond a critical time by forcing a jump as


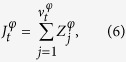


where 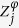, for *j* = 1, 2, …, are i.i.d. Gaussian random variables with zero mean and standard deviation *γ*^*φ*^, and 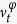 is a counting process defined such that, for time *r* ≤ *t*, 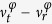 is a Poisson random variable with parameter *λ*^*φ*^(*t* − *r*).

**Figure 11:**
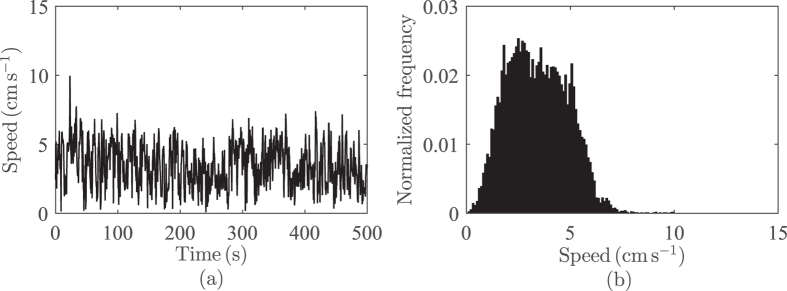
Sample fish speed time series (**a**) and distribution (**b**). Speed is modelled as a stochastic process that maintains a positive value.

Fish speed is found to vary about an average mean value while always staying positive (Fig. 11). We therefore model instantaneous change in fish speed, *υ*_*t*_(cm s^−1^) as a Cox-Ingersoll-Ross process^52^


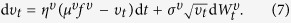


Here, *η*^*υ*^(s^−1^) is the adjustment rate controlling the rate at which fish speed reverts to its long term average value *μ*^*υ*^(cm s^−1^); *f*^*υ*^ = *f*^*υ*^(*ω*_*t*_, *φ*_*t*_) is a scaling function of *μ*^*υ*^; and 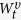 is a standard Wiener process weighted by the speed volatility *σ*^*υ*^(cm^1/2^ s^−1^). Different from Eqs (1) and (4), the Wiener process is weighted by the state variable, whereby we multiply the speed volatility by the square root of the speed. This allows for scaling the Wiener process proportionally to the instantaneous speed, ensuring that the speed is always nonnegative, provided that the remaining model parameters satisfy 2*η*^*υ*^*μ*^*υ*^ ≥ (*σ*^*υ*^)^2^. The scaling function *f*^*υ*^ is defined such that


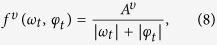


where *A*^*υ*^ (s^−1^) is a positive parameter to be determined. This functional dependence ensures that the speed decreases with the pitch and yaw rate, whereby the fish is expected to reduce its speed when undertaking a manoeuvre in either the vertical or horizontal plane. The stochastic differential equations Eqs (1), (4) and (7) define the proposed 3D model of zebrafish swimming.

Beyond the coupling through the function *f*^*υ*^ in Eqs (7) and (8), we assume that speed, pitch rate, and yaw rate processes are pairwise linearly correlated via the corresponding stochastic terms^77^. The proposed correlation structure is


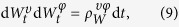



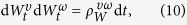


where 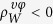 is the correlation coefficient between the speed and the pitch rate Wiener processes; 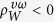 is the correlation coefficient between the speed and the yaw rate Wiener processes; Based on experimental data where absence or low correlation is observed between yaw and pitch rate, we hypothesize no correlation between pitch and yaw rates, and between the speed Wiener process and the pitch and yaw rate jumps, that is,


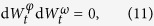



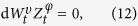



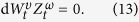


### Model calibration

The experimental dataset of 3D zebrafish swimming used to calibrate the model is described first, followed by our approach for parameter estimation, which is a combination of MLE and locally weighted regression. Next, we present the discrete form of the 3D fish swimming dynamics that is utilized to simulate trajectories. Finally, we detail the *in-silico* experiments that are used to validate and evaluate the proposed model.

### Experimental dataset for model calibration

**Figure 12:**
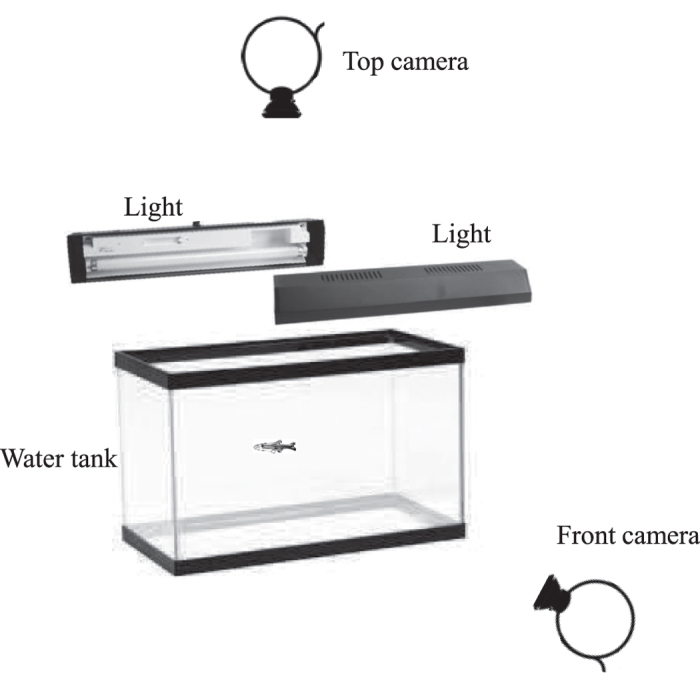
Illustration of the experimental setup. All sides, except the top and front, are covered with white contact paper.

We calibrate the model using a subset of the dataset collected as part of an earlier study^38^. Specifically, the data comprises of 3D trajectories of 10 experimentally naive zebrafish, filmed one at a time, in a 56 × 30 × 30 × cm compartment within a 76 × 30 × 30 × cm glass tank (Fig. 12) for 10 minutes each after 10 minutes of habituation. The 56 cm length compartment was separated from two side regions, 10 cm in length each, with a glass partition. The water depth in the tank was maintained at 26 cm. The zebrafish were filmed using two camera arranged to view the top and front side of the tank thus obtaining orthogonal views. The tank surface on four sides, where camera view was not required was covered with white contact paper for increased contrast during tracking. A custom tracking system was utilized to obtain horizontal and vertical trajectories of fish swimming which were then merged to form three-dimensional trajectory data. Additional details on the experimental procedure and the tracking algorithm can be found in ref. 38.

Fish 3D position data was filtered using a Daubechies wavelet filter^78^ to remove body movement due to tail-beat motion^54^. Similar to refs 43, 54, the curvature of the fish centroid trajectory was utilized to estimate the time trace of fish speed, pitch rate, and yaw rate. Stress-related measures, including freezing and thrashing, were computed automatically from the trajectory data^79^. A single trial where fish was found to freeze more than 70% of the time was discarded from the analysis, reducing the total number of trials to nine.

### Parameter estimation

The full 3D swimming model excluding boundary avoidance consists of 16 parameters denoted by Θ = (Ω, Φ, ϒ, *P*). These include: four independents parameters and two identical parameters each for yaw rate Ω = (*η*^*ω*^, *σ*^*ω*^, *γ*^*ω*^, *λ*^*ω*^, *k*_*W*_, *w*_*a*_) and pitch rate Φ = (*η*^*φ*^, *σ*^*φ*^, *γ*^*φ*^, *λ*^*φ*^, *k*_*W*_, *w*_*a*_) ; four independent parameters for speed ϒ = (*μ*^*υ*^, *η*^*υ*^, *σ*^*υ*^, *A*^*υ*^); and two correlation coefficients 
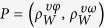
. Model parameters were estimated using MLE with a discrete time Euler approximation^80^. Denoting the time index by *k* = 1, 2, …, where *t*_*k*_ − *t*_*k*−1_ = Δ*t* (s), the discrete forms of Eqs (1), (4) and (7) are


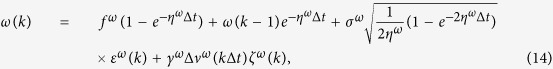



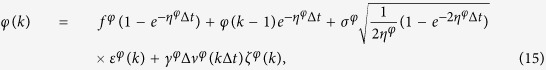



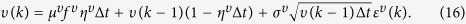


Similar to ref. 43, we hypothesize that a single jump occurs at most in a time step and accordingly, approximate the jump processes Δ*ν*^*ω*^(*k*Δ*t*) and Δ*ν*^*φ*^(*k*Δ*t*) by Bernoulli random variables with parameters *λ*^*ω*^Δ*t*, and *λ*^*φ*^Δ*t* respectively. In Eqs (14), (15) and (16), *ε*^*υ*^(*k*), *ε*^*φ*^(*k*) and *ε*^*ω*^(*k*) are all correlated Gaussian random variables.

We estimate the model parameters separately for fish swimming near the boundary and far from it. Specifically, we partition the fish trajectory data based on the distance from the wall, where position data further from 2 body-lengths (BL) ≃6 cm of the wall is considered to be not affected by the wall interaction response. This threshold of 2BL represents a rounded up value for measuring zebrafish peripheral activity^61^,^81^. Accordingly, we first estimate the fish state at time index *k* denoted by *X(k*) = [*ω(k*), *φ(k*), *υ(k*)]^T^, and at *k* − 1 without the boundary avoidance functions as 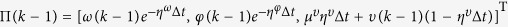. Using the above notation, the fish discrete state *X(k*) admits the following distribution:


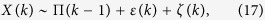


where 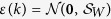 and 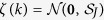, with **0** = [0, 0, 0]^T^ being the 3D null vector, and the covariance matrices for Wiener process, 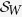, and jump processes, 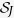, are


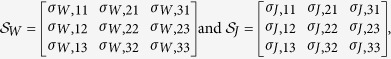


with


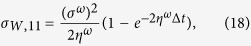



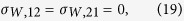



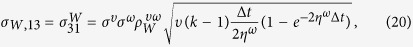



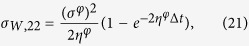



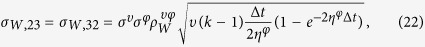



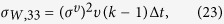


and


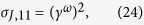



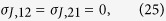



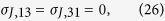



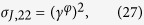



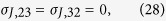



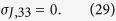


We introduce the system augmented covariance matrix 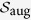, assumed positive definite, as


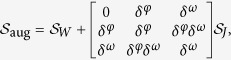


where *δ*^*φ*^ and *δ*^*ω*^ are equal to 1 when a jump occurs in the pitch or yaw rates respectively, and 0 otherwise. Using this augmented covariance matrix, the likelihood probability density function for the state of the fish at a given time index *k* is


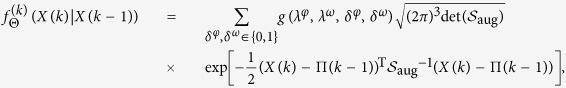


where 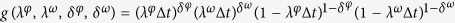 is the probability of a jump to occur in either pitch or yaw turn rate and det(•) is the matrix determinant. Considering a given 3D time series of zebrafish speed, pitch, and yaw turn rates 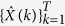, where *T* is the length of the dataset, model calibration is performed by maximizing the log-likelihood function^50^


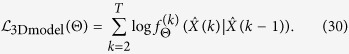


The MLE is implemented using the non-linear constraint algorithm available in the optimization toolbox of MATLAB (R2015a; MathWorks, Natick, Massachusetts, USA)^82^. To initialize and reduce the size of the large parameter set, three separate likelihood functions, corresponding to the three stochastic differential equations associated with, yaw rate, pitch rate, and speed, are utilized to estimate the values of a subset of the parameter set, consisting of the 11 independent parameters, with no correlation. Next, these values are used as initial values to maximize the 3D likelihood function in Eq (30). Table 2 lists the average value of estimated parameters. The full set of calibrated model parameters for each fish are available in the supporting information.

For wall interaction functions *f*^*ω*^ and *f*^*φ*^, regression fits are unable to capture two behaviours: wall interaction with low times-to-collision during which the fish performs a jump manoeuvre to prevent a collision, and wall following^28^. While simulating wall interaction with jumps require further complexity in the form of additional jump term, wall following is likely not captured due to the fit performed on trajectories. Specifically, trajectories will be rendered discontinuous due to specific threshold used to partition them into free swimming or wall interaction. In reality, this threshold may not be even a constant value. Accordingly, an instance of wall following may be captured in the form of two different collision avoidance manoeuvres if the fish swims beyond the assigned threshold even once.

During our simulations we found that we can achieve both these behaviours by increasing the value of *k*_*W*_. Accordingly, we select the value of *k*_*W*_ = 2.33 for *w*_*a*_ = 0.88 s^−1^. The parameter *A*^*υ*^ for the scaling function *f*^*υ*^ is estimated in two steps by utilizing a locally weighted regression to estimate the decay function of the absolute turn rate and the speed, and by applying a simple linear regression to approximate the decay rate which is averaged to 2.92 for the 9 fish datasets.

### Simulation of fish trajectories

Fish are simulated using Eqs (14), (15) and (16), that is, by Euler integrating the stochastic differential equations to obtain fish heading velocity at each time step. In particular, identifying fish position at *k* as 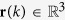 and its orientation in 3D through the azimuth *ψ(k*) ∈ [−*π, π*) and elevation 
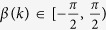
 in the tank frame (Fig. 9), the position and orientation at the next time index is


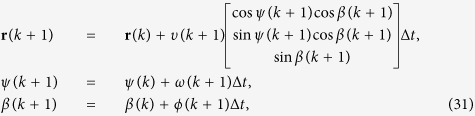


where *ω(k*), *φ(k*), and *υ(k*) are obtained as in Eqs (14), (15) and (16). The correlated Gaussian random variables *ε*^*υ*^(*k*), *ε*^*ω*^(*k*), and *ε*^*φ*^(*k*) are sampled on uncorrelated standard Gaussian random variables *ξ*^*υ*^(*k*), *ξ*^*φ*^(*k*), and *ξ*^*ω*^(*k*) with zero mean and unit standard deviation through^77^


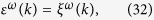



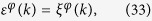



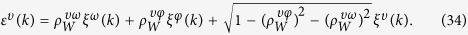


### *In-silico* experiments

Beyond demonstrating the accuracy of the proposed model on the experimental dataset in ref. 38, we consider an independent *in-silico* experiment addressing conditions which were not part of the dataset used for model calibration.

In the first computational experiment, a total number of 10 fish are simulated, each for 600 seconds with Δ*t* = 0.04 s within a 56 × 30 × 30 × cm bounded environment, replicating the experimental setup in ref. 83. Fish position is initialized so that all fish start at right top corner of the tank and the azimuth and elevation are oriented at a uniformly random direction. Model parameters are selected randomly to uniformly lie within one standard deviation of the average values (Table 2) obtained after model calibration on the real experimental data. This selection for the model parameters attempts at facilitating the process of replicating model predictions, and mitigating the dependence of inter-individual variability of the subjects on the predictions of ethological observables.

In the second computational experiment, we explore the role of tank dimensions (length, width, and depth) on zebrafish response. For a rectangular parallelepiped, we consider three different values for the depth (10, 20 and 30 cm), five different values for the tank width (5, 10, 20, 50 and 100 cm), and four different values aspect ratios (ratio of width to length: 0.25, 0.5, 0.75 and 1) width. Overall, we, simulate a total of 60 different tanks, spanning typical sizes considered in the literature (Table 1). We perform a total of 10 simulations for each tank geometry, similar to the first computational experiment. Each simulation is initialized as the first *in-silico* experiment. We set Δ*t* = 0.04 s and we simulate each fish for 12 minutes. Model parameters are randomized as in the first experiment.

### Analysis

The trajectories of the 10 simulated fish in the first experiment are utilized to generate a distribution of position and speed along the horizontal and vertical sections of the environment for visual comparison with the experimental data. All 10 simulated fish trajectories are then processed to compute ethological observables used for analysis in fish behaviour experiments^31^,^36^,^84^, such as: average speed; spatial preference along the water column scored from the time spent and number of entries in the top or bottom third of the tank; and the spatial preference along the tank length, measured from number of entries in either of the left or right third of the tank and the time spent therein. These observables are then compared with experimental data from^38^ using a non-parametric Kruskal-Wallis test^85^ which returns the chi-square statistics (*χ*^2^(·)) with degree of freedom (·), and the *p*-value.

For the simulations of the second experiment, fish behaviour is analysed in terms of the average speed, which is often reported in the technical literature as a measure of activity^60^,^86^ (Table 1). The correlation between the fish speed and each tank dimension is assessed using a generalized linear regression model (GLM)^87^. The same model is used to aggregate the simulation data and demonstrate the dependence of the speed on the overall tank surface area. The latter dependence is verified through comparison with experimental observations, which are also aggregated in terms of the total surface area. The total surface area accounts for the entire surface of the water, including the tank sides and the free surface. The experimental data listed in Table 1 is collated from 32 different zebrafish experiments, across a range of tank geometries and dimensions. We utilize the command *glmfit* of Matlab which returns the dispersion, the *F*-test statistics, and the *p*-value on the regression model assuming Gaussian error distribution. All the statistical tests are performed with a significance level set at 0.05.

## Additional Information

**How to cite this article**: Mwaffo, V. *et al*. *In-silico* experiments of zebrafish behaviour: modeling swimming in three dimensions. *Sci. Rep.* **7**, 39877; doi: 10.1038/srep39877 (2017).

**Publisher's note:** Springer Nature remains neutral with regard to jurisdictional claims in published maps and institutional affiliations.

## Figures and Tables

**Table 1:** Tank shape, dimensions, duration of the experiment, water volume, total surface area, and average speed from 32 different experiments reported in the literature.

Reference	Tank shape	Dimensions (cm)	Time (s)	Water volume (l)	Total surface (cm^2^)	Avg. speed (cm s^−1^)	Specific source
88	Rectangular parallelepiped	23.5 × 13.5 × 13.0	300	1.0	1408.8	6.2	Fig. 1d: 2D data available
Exp. 1^89^	Trapezoidal prism	27.9 × 6.4 × 15.2	300	1.4	1408.8	2.3	Fig. 1: speed estimated from 2D travelled distance
Exp. 2^89^	Trapezoidal prism	27.9 × 6.4 × 15.2	300	1.4	1408.8	2.2	Fig. 4: speed estimated from 2D travelled distance
37	Trapezoidal prism	27.9 × 7.1 × 15.2	360	1.5	1339.4	4.0	Figs 1 and 2: 3D data available
Exp. 1^58^	Trapezoidal prism	27.9 × 7.1 × 15.2	360	1.5	1339.4	2.2	Fig. 1a: speed estimated from 2D travelled distance
Exp. 1^59^	Trapezoidal prism	27.9 × 7.1 × 15.2	360	1.5	1483.7	2.7	Fig. 5b: speed estimated from 2D travelled distance
Exp. 2^59^	Trapezoidal prism	27.9 × 7.1 × 15.2	360	1.5	1339.4	3.9	Fig. 6b: speed estimated from 2D travelled distance
Exp. 1^81^	Trapezoidal prism	28.0 × 7.0 × 15.0	360	1.5	1337.6	2.5	Fig. 1c: 3D data available
Exp. 2^81^	Trapezoidal prism	28.0 × 7.0 × 15.0	1800	1.5	1337.6	4.4	Fig. 1d: 2D data available
Exp. 1^90^	Trapezoidal prism	28.0 × 7.0 × 15.0	360	1.5	1337.6	3.5	Fig. 1a: speed estimated from 2D travelled distance
60	Trapezoidal prism	28.9 × 7.4 × 15.1	900	1.5	1527.7	5.2	Fig. 5a: 2D data available
91	Trapezoidal prism	28.9 × 15.1 × 7.1	360	1.5	1527.7	4.7	Fig. 3: speed estimated from 3D travelled distance
40	Trapezoidal prism	27.0 × 7.0 × 15.0	360	1.5	1237.5	2.7	Fig. 5a: 3D data available
92	Trapezoidal prism	28.0 × 7.0 × 15.0	300	1.5	1337.6	4.0	Table 1: 2D data available
Exp. 3^81^	Cylinder	12.0 × 19.0	1200	2.0	893.0	2.0	Fig. 4: 2D data available
Exp. 3^90^	Cylinder	21.0 × 24.0	360	4.2	1483.7	6.0	Fig. 2a: 2D data available
66	Rectangular parallelepiped	30.0 × 10.0 × 15.0	60	4.5	1800.0	4.4	Fig. 1b: 2D data available
Exp. 1^93^	Rectangular parallelepiped	30.0 × 10.0 × 15.0	300	4.5	1800.0	3.9	Fig. 1c: 2D data available
Exp. 2^93^	Rectangular parallelepiped	30.0 × 10.0 × 15.0	300	4.5	1800.0	4.5	Fig. 2c: 2D data available
94	Rectangular parallelepiped	20.0 × 12.0 × 25.0	600	6.0	2080.0	5.9	Fig. 5: speed estimated from 2D travelled distance
Exp. 2^95^	Cylinder	21.0 × 24.0	360	8.3	1339.4	3.4	Fig. 2b: 2D data available
39	Rectangular parallelepiped	25.0 × 25.0 × 18.0	1200	8.8	2650.0	4.4	Fig. 1f: 3D data available
96	Parallelepiped	40.0 × 30 × 30	1800	36.0	6600.0	7.2	Table 2: 2D data available
97	Parallelepiped	54 × 30 × 30.0	600	24.3	5760.0	3.6	Fig. 8: 3D data available
98	Parallelepiped	50.0 × 25.0 × 30.0	1800	37.0	6940.0	4.3	Fig. 5: speed estimated from 2D travelled distance
99	Parallelepiped	50.0 × 30.0 × 25.0	3600	37.0	7000.0	5.8	Fig. 1: speed estimated from 2D travelled distance
100	Parallelepiped	56.0 × 30.0 × 30.0	900	40.6	7832.0	3.0	Fig. 6: 2D data available
101	Parallelepiped	56.0 × 30.0 × 30.0	600	43.7	7488.0	5.1	Fig. 4: 2D data available
102	Cylinder	90.0 × 10.0	300	63.6	15543.0	5.2	Fig. 4: 2D data available
38	Parallelepiped	56.0 × 30.0 × 30.0	600	43.7	7832.0	4.5	Table 1: 3D data available
103	Parallelepiped	120.0 × 120.0 × 20.0	300	144.0	33600.0	10.5	Fig. S1 (Supporting info): 2D data available
104	Parallelepiped	120.0 × 120.0 × 20.0	300	144.0	33600.0	9.3	Fig. 6: 2D data available

Values from control conditions were used to ensure that fish were not exposed to experiment-specific stimuli. Water volume may not match the tank volume due to lower water levels than tank height. Total surface area is taken as the sum of the area of all water surfaces except when water depth is not available, in which case the tank is assumed to be full (the same rule is used for the computation of the water volume). The dimensions for the rectangular parallelepiped and trapezoid are given in term of (length(cm) × width (cm) × depth (cm)) whereas for the trapezoidal tank shape, only the maximum length and the width at the top surface are reported. The cylinder shape is given in term of (diameter (cm) × height (cm)).

**Table 2:** Estimated model parameters from experimental data.

Parameter	Description	Mean ± std
*η*_*ω*_	Relaxation rate (yaw)	3.32 ± 0.46 (s^−1^)
*σ*_*ω*_	Turn rate variability (yaw)	3.00 ± 0.68 (rad s^−3/2^)
*γ*_*ω*_	Jump intensity (yaw)	3.27 ± 1.15 (rad s^−1^)
*λ*_*ω*_	Jump frequency (yaw)	0.42 ± 0.08 (s^−1^)
*η*_*φ*_	Relaxation rate (pitch)	3.72 ± 0.50 (s^−1^)
*σ*_*φ*_	Turn rate variability (pitch)	3.73 ± 0.63 (rad s^−3/2^)
*γ*_*φ*_	Jump intensity (pitch)	4.52 ± 0.97 (rad s^−1^)
*λ*_*φ*_	Jump frequency (pitch)	0.47 ± 0.02 (s^−1^)
*μ*_*υ*_	Long term mean speed	4.49 ± 1.26 (cm s^−1^)
*η*_*υ*_	Speed adjustment rate	0.26 ± 0.23 (s^−1^)
*σ*_*υ*_	Speed volatility	0.62 ± 0.27 (cm^1/2^s^−1^)
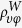	Correlation speed and yaw rate Wiener processes	−0.24 ± 0.67
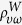	Correlation speed and pitch rate Wiener processes	−0.45 ± 0.62
*k*_*W*_	Intensity of the wall interaction	2.33
*w*_*a*_	Propensity to follow the wall	0.88 (s^−1^)
*A*^*υ*^	Speed decay coefficient	2.92 (s^−1^)
